# Squamous Cell Carcinoma In Situ—The Importance of Early Diagnosis in Bowen Disease, Vulvar Intraepithelial Neoplasia, Penile Intraepithelial Neoplasia, and Erythroplasia of Queyrat

**DOI:** 10.3390/diagnostics14161799

**Published:** 2024-08-16

**Authors:** Lucian G. Scurtu, Francesca Scurtu, Sebastian Catalin Dumitrescu, Olga Simionescu

**Affiliations:** 1 Faculty of Medicine, “Carol Davila” University of Medicine and Pharmacy, 050474 Bucharest, Romania; lucian.scurtu@drd.umfcd.ro (L.G.S.); sebastian.dumitrescu@ymail.com (S.C.D.); 2 Department of Dermatology I, Colentina Clinical Hospital, 020125 Bucharest, Romania; 3 Department of Obstetrics and Gynecology, Filantropia Clinical Hospital, 011132 Bucharest, Romania

**Keywords:** squamous cell carcinoma, Bowen disease, vulvar intraepithelial neoplasia, penile intraepithelial neoplasia, erythroplasia of Queyrat, skin cancer, HPV, lichen sclerosus, lichen planus, imiquimod, dermoscopy

## Abstract

Cutaneous squamous cell carcinoma (cSCC) is the second-most-prevalent malignancy in humans. A delayed diagnosis of cSCC leads to heightened invasiveness and positive surgical margins. Bowen’s disease (BD) represents an early form of cSCC and presents as a small erythematous, photo-distributed, psoriasiform plaque. Although certain dermoscopy features in BD are quite characteristic, histopathology remains the gold standard for diagnosis and provides a severity-scoring system that assists in guiding appropriate treatment strategies. The classification of precancerous lesions of the vulva and penis has undergone multifarious transformations due to variations in clinical and histopathological characteristics. Presently, erythroplasia of Queyrat is categorized as a clinical variant of penile intraepithelial neoplasia (PeIN). The diagnoses of vulvar intraepithelial neoplasia (VIN) and PeIN present significant challenges and typically necessitate one or more biopsies, potentially guided by dermoscopy. Aceto-white testing demonstrates a notably high negative predictive value for genital precancerous lesions. Histopathological examination represents the gold-standard diagnosis in VIN and PeIN, while p16 and p53 immunostainings alongside HPV testing provide crucial diagnostic clues. The histopathologic features, degree of differentiation, and associations with lichen planus, lichen sclerosus, and HPV guide the selection of conservative treatments or surgical excision.

## 1. Introduction

Cutaneous squamous cell carcinoma (cSCC) ranks as the second-most-prevalent neoplasm in humans following basal cell carcinoma, exhibiting an escalating incidence. The probability of cSCC onset is notably elevated, reaching up to 11% within the Caucasian demographic [1,2]. Additionally, cSCC stands as the second-leading cause of skin cancer fatality. Moreover, it is deemed the predominant contributor to skin cancer-related mortalities among the elderly, surpassing both melanoma and adnexal carcinomas. Notably, the propensity for metastasis is accentuated in elderly males and typically manifests 12–24 months after the treatment of the primary lesion. A delayed diagnosis of cSCC precipitates a 4.7-fold escalation in invasiveness and a three-fold increase in positive surgical margins [3,4,5].

Bowen’s disease represents an in situ cSCC, with a 3–5% risk of progressing into invasive SCC [6]. While SCC ranks as the most prevalent cancer of the vulva and penis, its incidence remains low in high-income nations. The treatment of invasive forms frequently results in disfigurement and inflicts a profound psychological toll on patients [7]. Consecutively, the timely diagnosis of penile (PeIN, penile intraepithelial neoplasia) and vulvar (VIN, vulvar intraepithelial neoplasia) incipient SCC remains a desideratum. Notably, glans and prepuce Bowen’s disease, historically referred to as erythroplasia of Queyrat, bears a transformation risk into invasive SCC of up to 33% [8,9].

This manuscript is intended to emphasize the significance of diagnosis in early cSCC and to address the principal diagnostic and management challenges associated with Bowen’s disease, VIN, PeIN, and erythroplasia of Queyrat.

## 2. Bowen’s Disease (BD)

BD typically affects individuals over 60 and is uncommon in those under 30 and black individuals. Immunocompromised individuals are at risk of developing BD at a younger age. Most studies indicate a slight female predominance. The incidence is higher among Caucasians (1.42/1000). In BD, the term “SCC in situ” denotes an intraepithelial lesion characterized by the clonal proliferation of atypical keratinocytes occupying the full thickness of the epidermis [10,11,12]. Lesions are generally solitary. The morphology of BD varies depending on the age of the lesion, the site of origin, and the degree of keratinization. BD is often referred to as the “lull before the storm”, indicating its role as a precursor to overt cSCC [13]. Progression to the invasive and metastatic form occurs over a long period in only 3% to 5% of cases [14].

The primary etiological factors of BD are exposure to ultraviolet light, immunosuppression, and human papillomavirus (HPV) infections. While BD commonly occurs in photo-exposed areas of the skin, it can also affect other regions [11]. Its pathogenesis encompasses various forms of radiation, including ionizing radiation, in addition to ultraviolet (UV) radiation [11,13]. Cumulative exposure to ultraviolet (UV) light radiation causes DNA damage and immunosuppression, which promotes the clonal expansion of cells with p53 mutations [15]. Furthermore, exposure to certain toxic substances, such as arsenic, and infection with oncogenic strains of HPV also contribute to the development of BD [11,13,16]. Most HPV-positive lesions regarding the skin in BD are commonly found on the distal extremities (such as periungual sites), with HPV type-16 often being detected [17,18].

Clinically, BD is classically described as a small, erythematous, scaly plaque that enlarges erratically over time [12]. The overlying scale can be white or yellow, either easily removable or adherent. When the scale is removed, it does not cause bleeding and exposes an erythematous, moist surface [11,19]. The margins of the lesion are well-defined, and the affected area is slightly raised above the normal skin level. The surface is generally flat but can sometimes become hyperkeratotic or crusted. The presence of an ulcer often indicates cSCC, except for palmar superficial lesions, which may result from repeated friction [19].

The primary differential diagnoses include infiltrating cSCC, superficial basal cell carcinoma, nummular eczema, condylomata acuminata, psoriasis, lichen planus, lichen simplex chronicus, lichen planus-like keratosis, porokeratosis, chronic discoid cutaneous lupus erythematosus, Paget disease and seborrheic keratosis [10,20].

**Figure 1:**
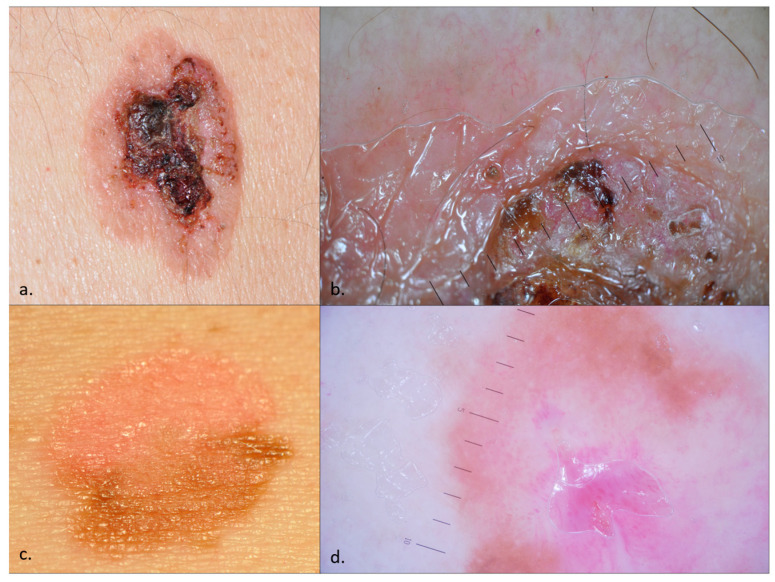
Bowen disease (BD)—clinical images (**a**,**c**) and dermoscopy (**b**,**d**). An ulcerated plaque with white scales, hematic crusts, and few vessels in the periphery mimicking a cSCC, whose pathology examination revealed an ulcerated, non-pigmented BD (**a**,**b**). While ulceration and crusting often characterize cSCC transformation, histopathological examination confirmed the absence of SCC in this lesion; An erythematous and pigmented plaque with fine scales, prominent glomerular vessels, and pigmentation, suggestive of fragmentarily pigmented BD (**c**,**d**).

Dermoscopy can assist in the diagnostic process, as the clinical presentations may pose significant diagnostic difficulties (Figure 1). The dermoscopic features of BD were first detailed by Zalaudek et al. in 2004. Since then, multiple studies have been conducted on the dermoscopic characteristics of BD, consistently identifying glomerular vessels, a scaly surface, small brown globules, and structureless grey to brown pigmentation as its primary features [15,17,21,22]. BD can be categorized into three types: (a) classic BD—irregular vascular pattern, whitish scaling, and a pinkish network; (b) pigmented BD—unstructured pigmentation, pigmented stripes, and crust formation; and (c) fragmentarily pigmented BD—a combination of characteristics from both classic and pigmented BD [14,23]. Pigmented BD manifests as heterogeneous brown plaques that may appear keratotic or verrucous. The most common differential diagnoses are seborrheic keratosis, solar lentigo, pigmented actinic keratosis, melanocytic nevus, and melanoma [16,23,24,25].

Histopathology remains the gold standard for diagnosis, revealing hyperkeratosis, parakeratosis, acanthosis with elongation and thickening of rete ridges, absence of the granular layer, and full-thickness keratinocyte atypia without breaching the dermo–epidermal junction [18,23]. The keratinocytes exhibit intense mitotic activity, pleomorphism, large nuclei, and loss of maturity and polarity, giving the epidermis a “windblown” appearance. Two types of giant cells in the epidermis are recognized: one with a keratinocyte engulfing a dyskeratotic cell, and another with multiple central nuclei surrounded by dyskeratotic tonofilaments. The dermis shows moderate lymphocytic infiltrates, the occasional vacuolization of upper dermal cells, and secondary amyloid deposition. BD may also involve sebaceous or mucinous metaplasia [11,26,27]. Histologic variants include psoriasiform, atrophic, acantholytic, epidermolytic, and other patterns like verrucous-hyperkeratotic, orthokeratotic, mucinous, sebaceous, papillated, irregular (highly pleomorphic), pigmented, pagetoid, and clear cell, some of which can be associated with HPV infection [14,23,26].

A grading system for BD (Table 1) helps stratify patients based on the severity of their condition. This scoring system allows for a standardized assessment of BD severity and aids in guiding appropriate treatment strategies and follow-up care. Adjustments and validations by clinical studies may further refine this system to ensure its efficacy and accuracy [11,17,19,21,23,25,28,29,30,31,32,33].

BD is characterized by distinct immunohistochemical profiles that aid in its differentiation from other dermatological conditions. Keratinocyte nuclei in BD show a diffuse pattern of staining for proliferating cell nuclear antigen (PCNA), and cytokeratin 10 (CK10) is universally expressed. The overexpression of p16 reflects disrupted G1/S checkpoint control, useful for distinguishing BD from actinic keratosis (AK) and seborrheic keratosis. A distinct p53 and p16 staining pattern in basal epidermal keratinocytes is instrumental in distinguishing BD from AK. Unlike BD, Paget’s disease expresses CK7, while BD of the nipple stains positive for cytokeratin 5/6 and negative for CK7 [34,35,36]. Markers such as Ki-67 and p27 provide additional diagnostic clarity; Ki-67 shows a diffuse pattern in BD, differentiating it from AK, and p27 indicates cellular latency, distinguishing BD from cSCC [36,37]. Positive immunostaining for lumican and the expression of CK14, associated with tumor progression, further assist in the histopathological evaluation [35].

## 3. Vulvar Intraepithelial Neoplasia (VIN)

Throughout the years, the terminology associated with vulvar precancerous lesions has witnessed diverse transformations attributable to disparities in clinical and histopathological aspects. A consensus on the terminology was achieved by the International Society for the Study of Vulvovaginal Diseases (ISSVD) in 2015. The evolution of the terminology for vulvar precancerous lesions dates back to the description of erythroplakiform dyskeratosis in 1922 [38,39]. Subsequent nomenclature variations included Bowen dermatosis (1929), carcinoma in situ (1943), vulvar atypia (1972), Bowenoid atypia (1973), hyperplastic dystrophy with atypia (1976), and Bowenoid papulosis (1979) [40].

The term vulvar intraepithelial neoplasia (VIN) was formalized in 1982 and underwent further subclassification over time [41,42]. Initially, VIN was categorized as squamous VIN, encompassing subtypes 1–3 representing varying degrees of atypia, along with non-squamous VIN of the melanoma or Paget’s disease type. The designation “differentiated VIN” was introduced in 1986, based on histopathologic considerations. In 2004, the ISSVD Group redefined the classification of VIN into two groups, delineated by histopathologic and pathogenic aspects: VIN of the usual type, induced by HPV, subdivided into basaloid, warty, mixed, and VIN of the differentiated type, non-HPV-infected. VIN 1 disappears due to a skin reaction to HPV infection rather than a precancerous lesion [41].

In 2012, the grading of VIN from 1-2-3 was replaced by a standardized terminology, which replaced VIN, CIN (cervical intraepithelial neoplasia), and PeIN (penile intraepithelial neoplasia), with the term “SIL” (squamous intraepithelial lesion) [43]. This term characterizes the group of precancerous lesions of the lower genital tract and anogenital tract caused by HPV. The SIL system comprises LSIL (low-grade SIL) and HSIL (high-grade SIL) [44,45]. There have been multiple debates focusing on the pathological significance of vulvar LSIL lesions, with arguments against their treatment. From 2014 onwards, diagnostic and treatment protocols have been premised on classifying vulvar precancerous lesions, encompassing LSIL, HSIL, and differentiated VIN (simplex) [40]. In summary, vulvar LSIL (VLSIL, formerly known as VIN 1), denotes flat condyloma, an HPV-related vulvar lesion. Vulvar HSIL (VHSIL, previously referred to as VIN 2 and VIN 3) signifies the usual type of VIN, whereas differentiated VIN indicates vulvar lesions unrelated to HPV, often associated with vulvar dermatoses, particularly lichen sclerosus.

Multiple cohort studies have underscored an elevated prevalence of VHSIL, with an incidence of 3.8 per 100,000 women per year. This rate surpasses the incidence of differentiated VIN. Concurrently, an increased manifestation of VHSIL has been documented, escalating from 2.39 to 3.26 per 100,000 females between 1991 and 2011. These findings underscore the significance of this issue from a public health perspective [46]. This variance is partly attributable to the heightened frequency of HPV infections before the era of widespread vaccination, the relatively young age of women falling into this category, and the enhanced discernibility upon the manifestation of a vulvar lesion [47]. In contrast, diagnosing differentiated VIN poses greater challenges for both clinicians and pathologists, as it is more commonly observed in older women with concomitant lichen sclerosus, with an average age of 68 years [48]. According to Virnig et al., there was a 411% increase in the prevalence of vulvar carcinoma in situ among women aged 40–49 from 1973 to 2000 [49], with the highest reported frequency comprising women aged 20 to 35 years [50]. There has been no apparent change in the incidence of VSCC over time. This stability can be attributed to the increased early detection of pre-invasive lesions and the subsequent expedited treatment [51].

Differentiated VIN (dVIN) has a higher propensity to progress to vulvar SCC than HSIL (43.2%, respectively 9.7%). Moreover, patients with dVIN pose a higher risk of developing recurring vulvar SCC [46,52]. VHSIL is linked to the presence of high-risk HPV in 80% of cases. Among these cases, HPV 16 is predominant, accounting for 80%, followed by HPV 33, which is present in 6% of cases [53]. In another study, the majority of LSILs were associated with low-risk HPV types 6 and 11, comprising over 90% of cases [54].

Immunosuppression is a critical factor in the development of VHSIL, with HPV playing a pathogenic role that is similar to other preinvasive lesions of the lower reproductive tract, including the cervix and vagina [55]. Additionally, multiple low-risk and high-risk HPV strains can coexist in the same lesion [44,56,57]. Vulvar condyloma acuminatum is primarily attributed to low-risk HPV 6 and 11 and is not considered to pose a theoretical risk of neoplastic development. Nevertheless, several studies have indicated an indirect association with anogenital, head, and neck cancers due to co-infection with high-risk strains [57,58]. Individuals with compromised immune systems may rarely experience a malignant transformation of anogenital warts, especially in Buschke–Löwenstein tumors [59,60].

Additional risk factors identified in the literature for VHSIL encompass smoking, the number of sexual partners, and immunosuppression resulting from HIV infection, coinfection with herpes simplex virus (HSV), human T-lymphotropic virus type I (HTLV-I) infection, or chronic illnesses [61,62,63,64]. Upon diagnosing a vulvar lesion, it is essential to conduct a comprehensive evaluation that encompasses not only the vulva but also the anal region, vagina, and cervix, as these areas frequently coexist. For instance, in 60% of cases with VaIN (vaginal intraepithelial neoplasia) or VIN, synchronous or pre-existing CIN is identified. It is noteworthy that 10% of patients with CIN 3 may exhibit squamous intraepithelial neoplasm in other sites. Additionally, HPV is detected in more than 94% of VaIN 2/3. These considerations bear significant importance in understanding the recurrence of lesions [65,66,67,68].

The distinct etiology and epidemiology of the two forms of intraepithelial neoplasia also signify their differing pathogenesis. VHSIL is linked with HPV lesions and exhibits a heightened incidence of other anogenital cancers due to their common embryologic origin [67,68,69,70]. Consequently, VHSILs are frequently found to be multicentric and multifocal, thereby accounting for metachronous and synchronous occurrences of cancer across the vulva, anus, cervix, and vagina [70].

Lesions resulting from high-risk HPV are attributable to an immune escape mechanism, as previously shown in other sexually transmitted disorders [71]. This mechanism involves HPV replication within host cells without the production of viral antigens that would typically trigger an immune response. Hence, cytolysis and inflammation are absent due to reduced levels of IFN type-1 induced by the E6 and E7 oncogenes [72]. The downregulation of cytotoxic T cells in the local environment is attributed to the decreased activity of HLA class I, facilitated by the E5 protein. This dynamic allows for the immune system to tolerate the silent viral replication, along with the tumor microenvironment, ultimately contributing to the progression of preneoplastic lesions [73,74]. Less than 5% of VHSILs progress to vulvar cancer, while approximately 9.7% of women under 35 years of age who have been treated for a previous VHSIL develop this condition [70,75].

**Figure 2:**
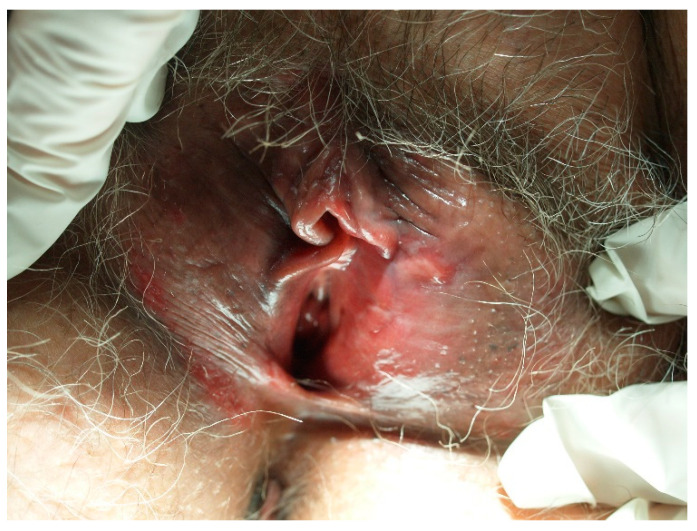
Vulvar intraepithelial neoplasia (VIN) developed on lichen sclerosus in a postmenopausal patient.

The majority (80%) of keratinized vulvar cancers are preceded by differentiated vulvar intraepithelial neoplasia (dVIN). It is believed that dVIN develops as a result of aberrant cellular functions instigated by genetic oxidative stress. dVIN lesions typically manifest as single, site-specific growths and are often linked to persistent inflammatory conditions, such as lichen sclerosus or lichen planus (Figure 2). Notably, these lesions exhibit an aggressive progression toward SCC. The association of dVIN with lichen sclerosus frequently leads to poorly defined lesions that are resistant to topical therapies. Lichen sclerosus associated with dVIN often precedes the development of vulvar cancer, with the risk of transformation increasing with age [52,61,62,63,76,77,78]. Gallio et al. observed that of the 76 patients with dVIN, more than 80% presented associated lichen sclerosus, while no instances of lichen planus were reported. This observation is in line with the conflicting data on the correlation between lichen planus and VIN [52].

Alongside the conventional TP53 mutagenic pathway [79], the presence of NOTCH1 mutation leading to the loss of the tumor suppressor function and the involvement of the HRAS oncogene in the RTK/RAS/PI(3)K pathway have been demonstrated to contribute to the pathogenesis of dVIN cancers through somatic mutations and aberrant cell proliferation [48,70,80,81].

The clinical presentation of VIN lesions is diverse and lacks pathognomonic features. The presence of abnormal cervical cytology or a positive high-risk HPV test necessitates a thorough examination of the vulvo-perianal region, the pubis, and the lymph nodes, as screening tests are not standardized. VIN lesions may be asymptomatic. However, patients frequently report experiencing superficial dyspareunia or vulvar pain, along with symptoms such as burning, itching, discharge, or bleeding, which may indicate the potential invasion of the lesion. In clinical practice, it is not uncommon to observe various lesion patterns within a single patient. These lesions may exhibit pigmentation and appear white, gray, red, warty, flat, or raised. During the examination, it is imperative to meticulously note the number, shape, topography, size, thickness, and color of each lesion [48,82,83], as presented in Table 2 [83,84,85,86].

dVIN lesions are often found in patients with a history of vulvar cancer or the proximity of a vulvar SCC. The lesions can be recognized as white or gray discolorations with a bumpy surface, elevated nodules, or faint thick plaques [48].

Cytology obtained directly from vulvar lesions is hindered by the thick keratinization layer of the vulvar skin, leading to inefficiency. Despite its low specificity, vulvar aceto-whitening demonstrates nearly 100% sensitivity. In a study involving 344 women, Likes et al. established that vulvoscopy, combined with the application of 3–5% acetic acid, exhibits a notably high negative predictive value [87]. This implies that the absence of an aceto-whitening lesion correlates with the absence of a precancerous lesion. Nevertheless, the application of acetic acid to the vulvar area should be performed exclusively by experienced professionals.

In instances of multifocal lesions, vulvar mapping is deemed essential, and dermoscopy serves to assist the clinician in minimizing redundant biopsies. Consequently, efforts are being made to establish dermatoscopic criteria for successful differential diagnosis, both in distinguishing between the severity grades of the lesions and in differentiation from other conditions. In the absence of standardized criteria for assessing the dermatoscopic appearance of vulvar lesions, various authors have published case series utilizing dermoscopy to advocate for the adoption of this practice (Table 3).

The differential diagnosis of primary dermoscopy structures/features in VIN is of utmost importance: dotted vessels (extramammary Paget disease, vulvar psoriasis), glomerular vessels (BD), dotted and linear vessels (vulvar lichen sclerosus), cerebriform pattern (vulvar seborrheic keratosis) and parallel pigmented dots with well-demarcated borders (pigmented BD, lentigo) [91,92,93,94,95]. However, there is currently no consensus regarding the dermoscopy diagnosis of VIN. The histopathology examination represents the gold-standard diagnosis in VIN. Table 4 and Table 5 present the pathology features of VHSIL and dVIN [48,82,85,96,97,98].

## 4. Penile Intraepithelial Neoplasia (PeIN)

The incidence of penile cancer in Europe and North America is low (<1/106). Most forms of penile cancer are SCC, classified into 12 histopathological forms, among which the keratinized, conventional form predominates [99,100]. Penile SCC is associated with lack of circumcision, low socioeconomic status, smoking, phimosis, poor hygiene, and sexually transmitted infections, especially human immunodeficiency virus, and human papillomavirus [101,102]. Its most common localizations are the glans, mucosal surface of the foreskin, and coronal sulcus of the penis [103].

The terminology associated with in situ penile SCC is intricate. Presently, the term erythroplasia of Queyrat (EQ) is used synonymously with Bowen’s disease of the penile mucosa and holds heightened clinical significance. However, it should be differentiated from the BD of the penile shaft and other non-mucosal genital regions, which should be stratified according to Table 1. Over time, the classification of penile lesions has undergone significant revisions, with the contemporary preference being the concept of penile intraepithelial neoplasia (PeIN), akin to cervical intraepithelial neoplasia (CIN) [104,105]. Consequently, EQ/Bowen’s disease of the penile mucosa is currently regarded as a form of PeIN. Although some authors classify Bowenoid papulosis as a PeIN, it typically follows a benign course and may warrant classification as a distinct entity [106,107].

Lichen planus and lichen sclerosus are the primary pre-existing penile dermatoses strongly associated with PeIN. Additional risk factors consist of preputial diseases (phimosis, paraphimosis, and adherent prepuce), prior penile surgery, use of immunosuppressive drugs, presence of genital warts, history of organ transplantation, and phototherapy. Notably, the uncircumcised state remains the predominant risk factor for PeIN [108,109].

Any enduring brown-gray, red, or white penile papules, plaques, or macules should prompt the suspicion of PeIN. PeIN typically presents as an asymptomatic condition, although pain, bleeding, crusting, and pruritus may manifest. In circumcised men, PeIN may exhibit scales atop a fully keratinized glans. Clinical examination of a suspected penile lesion includes the application of 3–5% acetic acid, although its specificity for PeIN is limited, as only 20% of PeIN lesions yield a positive reaction to acetic acid [110,111].

In clinical practice, it is often difficult to differentiate different forms of PeIN from other dermatoses, and the diagnosis is even more difficult in the case of PeIN lesions developed on pre-existing, chronic, penile lesions. Infectious (herpes progenitalis, fungi, syphilis), inflammatory (seborrheic dermatitis, lichen planus, Zoon’s balanitis, fixed drug eruption, psoriasis, balanitis circinate, etc.), and neoplastic (e.g., extramammary Paget’s disease) dermatoses have to be ruled out [112,113,114,115].

**Figure 3:**
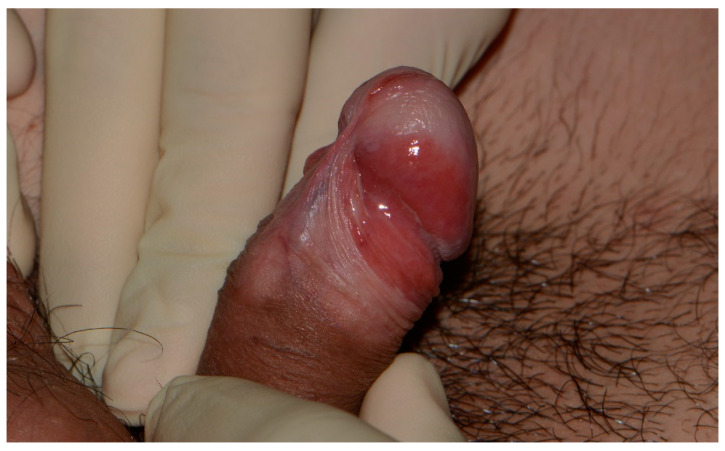
Erythroplasia of Queyrat involving the glans, coronal sulcus, and prepuce. Glans etiolation, frenulum sclerosis, and the waxy pallor of the distal prepuce are suggestive of an associated lichen sclerosus.

EQ typically manifests as slowly progressive, well-defined, slightly elevated, erythematous plaques on the glans penis, coronal sulcus, and prepuce, predominantly affecting uncircumcised men. Histopathological examination often reveals penile intraepithelial neoplasia (PeIN) and may be managed through local resection, ablative laser treatment, and topical agents such as 5-fluorouracil and TLR-7 agonist (imiquimod). Glansectomy is associated with the lowest recurrence rate [116,117,118]. Unlike the discernible appearance of EQ (Figure 3), the emergence of PeIN on pre-existing skin lesions like lichen planus and lichen sclerosus may pose diagnostic challenges.

Genital lichen planus has a higher incidence in men, affecting 25% of individuals diagnosed with lichen planus. It manifests with annular (most commonly on the glans), reticular, erosive, bullous, and atrophic appearances. Progressive developments may include PeIN, penile SCC, stenosis, and synechiae [119]. Noteworthy changes in a lichenoid clinical lesion such as ulceration, pain, or the growth of a mass may indicate the potential development of PeIN or SCC [120].

Penile lichen sclerosus predominantly affects men aged 30–40, with an estimated incidence rate of up to 0.3%. It commonly involves the meatus, foreskin, penile shaft, and glans penis and is frequently a disease of the uncircumcised, due to the chronic exposure of the epithelium to urine (urinary dribbling/microincontinence). Initially, it manifests as an erythematous plaque, progressing to atrophy and depigmentation, often presenting a pearly appearance, and demonstrating proclivity toward scarring and erosion [121]. The contentious association between lichen sclerosus and HPV and *Borrelia burgdorferi* infections is subject to ongoing debate. Studies have not found a significant correlation between lichen sclerosus and HPV infection, with the association with HPV-16 considered more incidental than causal. Additionally, most research did not yield evidence of Borrelia DNA in skin biopsy specimens. Treatments for penile lichen sclerosus encompass surgical interventions and conservative modalities such as topical steroids, intralesional steroids, and platelet-rich plasma injections. Of note, it recurs in skin grafts. Left untreated, lichen sclerosus may advance to PeIN or SCC. Its malignant transformation risk is variable, estimated between 4 and 13.4%, while 12% of penile SCC is due to lichen sclerosus. Notably, differentiated PeIN prevalence surpasses that of undifferentiated subtypes among lichen sclerosus patients [121,122,123]. Pseudoepitheliomatous keratotic and micaceous balanitis (PEKMB) represents a chronic and unstable form of lichen sclerosus with a significant potential to develop into an SCC. It clinically presents as silvery scales on the glans in patients with an undiagnosed or treatment-refractory lichen sclerosus [124].

HPV-associated PeIN is associated with the viral genome integration into the human genome, which leads to oncogene overexpression and malignant transformation [125], as in VIN. HPV types 6 and 11 are more frequent in condyloma lesions and subtypes 6, 11, and 18 in the PeINs. However, less than 1% of the penile HPV progress to PeIN, with a median time from infection of 12.7 months [126].

**Figure 4:**
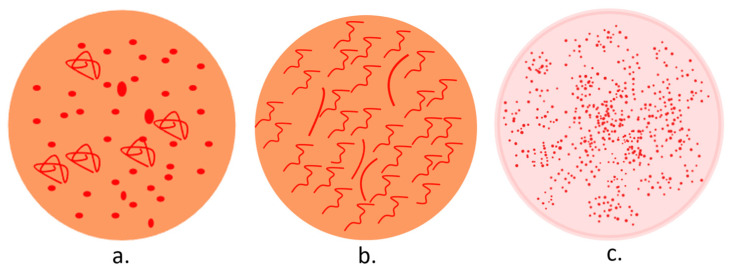
Dermoscopy patterns in PeIN (**a**), Zoon balanitis (**b**), and penile psoriasis (**c**).

A dermatoscopic examination of any penile lesion suspected to be PeIN can provide numerous diagnostic clues. In addition, it can facilitate a dermoscopy-guided biopsy and differential diagnoses. In dermoscopy (Figure 4), PeIN displays an orange structureless area and a vascular pattern, comprising nonhomogeneous dotted (predominantly) and glomerular vessels. Its main differential, Zoon balanitis, presents an orange structureless area and linear-curved vessels, while glans psoriasis reveals diffuse and homogeneous dotted vessels on an erythematous background [127,128,129].

From a histopathologic point of view, PeIN represents a dysplastic modification of the epithelium that does not affect the basement membrane, which remains intact [106]. Nowadays, PeIN is classified into differentiated and undifferentiated morphologic subtypes, which correlate with HPV infection status [130]. Table 6 displays the main PeIN subtypes according to pathology features and HPV infection [112,124,130,131,132,133].

## 5. Current Treatments in BD, VIN, and PeIN

The effective management of BD involves the utilization of a diverse range of treatment modalities customized to the specific characteristics of the lesions, the patient’s health condition, and their individual preferences. Commonly employed topical therapies, such as 5-fluorouracil and imiquimod, demonstrate efficacy in targeting atypical keratinocytes. Furthermore, curettage, electrodesiccation, and cryotherapy are viable options for treating small, superficial lesions, while surgical excision with histological margin control stands as the gold standard for ensuring the complete removal of larger or recurrent lesions. Laser therapy is a non-invasive alternative suitable for cosmetically sensitive areas, while radiotherapy is specifically reserved for challenging lesion locations or patients ineligible for surgery [29,32,33,134,135,136].

The objective of VIN treatment is the prevention of recurrent invasive cancer and the enhancement of the patient’s quality of life through the amelioration of symptoms and functionality of the vulvar region. Additionally, careful consideration of a low-risk treatment approach is essential [137]. The treatments for dVIN and VHSIL share similarities, yet each possesses distinct nuances in their methodologies. The loop electrosurgical excision procedure and cold-knife surgical excision are highly recommended for both VIN subtypes, preferably with 4–5 mm deep and lateral margins. Considering the higher recurrence rate in dVIN, wide surgical excision becomes warranted. Following dVIN excision, topical high-potency steroids should be applied to the remaining lichen sclerosus lesions. As for alternative therapies, topical imiquimod and 5-fluorouracil are particularly relevant for VHSIL [70,138,139,140]. The presence of HPV-16 infection, together with small lesions, correlates with a favorable therapeutic outcome and a reduced likelihood of recurrence after topical imiquimod therapy. Conversely, multifocal lesions and dVIN are indicative of a less-favorable response to imiquimod treatment [141,142]. Of note, CO_2_ laser therapy should be avoided on hair-bearing skin in VIN patients [142].

In the management of PeIN, circumcision plays a significant role, as it facilitates the removal of potentially neoplastic tissue and may enhance the subsequent application of topical treatments. Local therapy options include topicals like 5-fluorouracil, imiquimod, cryosurgery, and CO_2_ lasers. Combining local treatments has been associated with lower recurrence rates than monotherapy, akin to other precancerous/cancerous dermatoses. Surgical excision with penile sparing is recommended for relapsing/non-responsive lesions [143,144,145]. However, the surgical management of PeIN may result in a recurrence in up to 30% of cases. Circumcision commonly resolves all preputial PeIN. Following surgical intervention for PeIN of the glans, recurrence rates are as follows: 25% after wide local excision, 4% after Mohs surgery, 5% after total glans resurfacing, and 10% after glansectomy [146]. In a study conducted by Kravvas et al., it was revealed that among 345 patients diagnosed with PeIN, 8.7% exhibited concomitant SCC. Furthermore, 58% of these patients were found to have an association with HPV, while 12% showed indications of lichen sclerosus. Interestingly, 29.4% of the patients displayed a co-occurrence of both HPV and lichen sclerosus. The study’s findings suggest that a sole reliance on topical treatments is unsatisfactory, with less than 15% of patients demonstrating the potential for successful treatment using topical agents alone [147].

## 6. Conclusions

The clinical, histological, and immunohistochemical profile of BD, including markers such as PCNA, CK10, CK14, p16, p53, Ki-67, and p27, provide critical insights for its diagnosis and differentiation from other skin lesions. Diagnosing VIN and PeIN poses considerable challenges and typically entails one or more biopsies, potentially guided by dermoscopy and aceto-white testing. The vascular pattern against an orange background facilitates the differentiation of PeIN from other penile dermatoses, whereas dermoscopy in VIN often exhibits less distinct characteristics. Immunohistochemical and HPV tests assist in guiding the choice of therapies for the treatment of VIN and PeIN. The management of these cases is further complicated by the frequent association with lichen sclerosus, which perturbs the local anatomy and is linked to an elevated risk of recurrence following surgical excision.

The early diagnosis of BD, VIN, and PeIN is crucial due to their potential for malignant transformation and significant impact on patient outcomes. Detecting these early SCCs at an initial stage enables timely and effective treatment, reducing the risk of progression to invasive SCC. Early intervention not only improves prognosis but also minimizes the likelihood of metastasis and associated complications.

## Figures and Tables

**Table 1:** Bowen’s disease (BD) severity scoring system.

**Clinical Parameters (0–10 Points)**	
**Lesion Size**	Small (≤1 cm): 1 point Medium (1–2 cm): 2 points Large (>2 cm): 3 points
**Number of Lesions**	Single lesion: 1 point Multiple lesions: 3 points
**Location**	Non-cosmetically sensitive areas (e.g., trunk, limbs): 1 point Cosmetically sensitive areas (e.g., face, hands, genitalia, penile shaft): 2 points
**Macroscopic aspect**	Well-demarcated plaque: 1 point Irregular borders: 2 points
**Pathology features (0–20 points)**	
**Epidermal Involvement**	Partial thickness: 1 point Moderate: 2 points Severe: 3 points
**Dermo-Epidermal Junction**	Intact: 1 point Disrupted: 2 points
**Inflammatory Infiltrate**	Minimal: 1 point Moderate: 2 points Severe: 3 points
**Dermo-Epidermal Junction**	Intact: 1 point Disrupted: 2 points
**Keratinocyte Atypia**	Minimal: 1 point Moderate: 3 points Severe: 5 points
**Hyperkeratosis**	Mild: 1 point Moderate: 2 points Severe: 3 points
**Parakeratosis**	Absent: 0 points Present: 2 points
Low Risk (5–10 points): Typically managed with topical therapies or destructive modalities. Close follow-up is recommended. Moderate Risk (11–20 points): May require more aggressive treatment, including surgical excision or combination therapies. Regular monitoring for recurrence or progression. High Risk (>20 points): Often necessitates comprehensive treatment approaches, potentially involving multi-disciplinary care. Close and frequent monitoring for signs of progression to invasive cSCC.	

**Table 2:** Clinical presentations of VIN and main differentials with vulvar lesions.

Primary Lesion	Characteristics	Differentials
**Erythematous**	- Decrease in the thickness of the epithelium - Increased vascularity - Thinning of the epidermis - Ulceration - Inflammatory vasodilatation - Neovascularization	- Eczema - Candidiasis, folliculitis - Psoriasis, lichen planus - Paget’s disease, vulvar cancer
**White/gray lesions**	- Superficial keratin layer - Any degree of depigmentation - Relative tissue avascularity - Reaction to acetic - Maceration of keratin due to increased moisture of the vulva (especially when the keratin layer is thick)	- Lichen sclerosus - Bowenoid papulosis
**Depigmentation**	- Absence or loss of melanin	- Vitiligo
**Brown/black**	- Increased concentration of melanin - Intra-epithelial and/or intradermal melanin - Amplified synthesis of melanin in epidermal melanocytes - “Melanin incontinence”—phagocytosis of the excess of melanin in the papillary dermis	- Trauma - Estrogen topicals for vaginal atrophy - Hormonal contraception - Nevi - Lentigo - Mucosal macula - Melanoma
**Violet-reddish**	Vascular tissue	- Angioma - Choriocarcinoma

**Table 3:** Dermoscopy characteristics of VIN.

Author	Clinical Appearance	Dermoscopy	Pathology
De Giorgi et al. (2023) [88]	(1) Reddish shiny lesion with clear margins.	Homogeneous erythematous area (entire lesion) does not disappear under pressure. Vascular pattern: regular glomerular vessels.	VIN
	(2) Whitish erythematous, dyschromic lesion.	Non-compact white areas, homogeneous with erythematous areas to be seen transparently.	VIN
	(3) Whitish dyschromic lesion.	Compact milky-white areas (entire lesion).	VIN
	(4) Pigmented lesion with a color spectrum from light brown to dark brown.	Diffuse pigmentation, linear distribution with delimited translucent whitish areas, and no pigmented network.	Pigmented VIN
	(5) Red to white non-pigmented lesion.	Atypical vascular pattern with a variable red-to-white background.	VIN
Barisani et al. (2017) [89]	(1) Vulvar hyperkeratotic, warty, papillomatous plaque.	Papillomatous, hyperkeratotic scales with an erythematous center and whitish peripheral borders, white homogeneous keratotic areas, pink-to-red areas, and erosions. Vascular pattern: dotted and glomerular vessels.	VIN, HPV 33
	(2) Hyperkeratotic, multifocal vulvar plaque.	White hyperkeratotic, vegetating structures adjacent to smooth, pink-to-red areas. Vascular pattern: dotted and glomerular vessels.	VIN, HIV
	(3) Erythematous, asymptomatic vulvar plaque.	Uniform whitish background with pink areas. Vascular pattern: curvy and short serpentine vessels.	dVIN History of: surgical excision of an invasive vulvar SCC vulvar lichen sclerosus (VLS)
	(4) Pigmented lesion, flat, well-demarcated, with a rough surface/	Light-brown background with hyperpigmented, cerebriform structures and well-defined borders; parallel pigmented dots.	VIN, HPV 18
Rao et al. (2023) [90]	Hyperkeratotic verrucous brown plaque with few erythematous eroded areas	- Finger-like pattern - Knob-like pattern - Dotted and glomerular vessel at the center of a white halo. - Mosaic pattern with white reticular structures and glomerular vessels within them. - Few hyperkeratotic areas as white scales. - Brown to gray pigmented dots.	VHSIL
Ronger-Savle et al. (2011) [91]	Nonmelanocytic lesions	- Cerebriform pattern - Vessels	VIN

**Table 4:** VIN histopathology characteristics.

VIN	Microscopic Findings	Immunohistochemistry	HPV Association
**dVIN**	- Acanthosis, parakeratosis, irregular elongation, and anastomoses of the rete ridges. - Dyskeratosis, squamous pearls, and keratinization with eosinophilic cytoplasm due to the accumulation of intracellular keratin, gives a hypereosinophilic appearance. - Moderate to marked cellular atypia is only confined to the basal and parabasal cells of the epidermis. - Basal layer atypia: nuclei enlargement, irregular nuclear contour (angulation), basal cell proliferation, coarse chromatin/open vesicular nuclei, prominent nucleoli, and scattered mitoses. - Prominent intercellular bridges (spongiosis or acantholysis) in the lower third of the epithelium. - Rare extension to skin appendages.	**P16**: Usually negative. Few cases display a non-blocklike pattern limited to the lower epithelial half. **P53**: Usually positive (>80% dvin with TP53 mutation), most prominent in the basal layer, with suprabasal extension.	Not HPV-driven.Lichen-sclerosus associated.
**VHSIL**	- Full-thickness atypia: proliferation begins at the basal layer and involves the full thickness of the epithelium. - Architectural disarray (“wind-blown” pattern). - Acanthosis, hyperkeratosis, parakeratosis, club-shaped rete ridges. - Atypical basal cells with large, dark nuclei and basophilic cytoplasm, may develop an eosinophilic cytoplasm approaching the surface epithelium. - Multiple mitoses, increased nuclear-to-cytoplasmic ratio, hyperchromasia, pleomorphism, apoptotic bodies. - Frequent extension to skin appendages (follicular epithelium, sebaceous glands).	**P16**: Usually positive, in a block-like pattern (diffuse, strong, and continuous). **P53**: Usually negative.	HPV-driven.

**Table 5:** Morphologic subsets of dVIN and VHSIL.

dVIN		VHSIL		
Keratinizing (>40% of the Epithelium Express Maturation)	Non-Keratinizing (<40% of the Epithelium Express Maturation)	Warty	Basaloid	Mixed
Four subtypes: - Hypertrophic > 0.6 mm - Traditional - Acantholytic - Subtle 0.1–1.5 mm - Atrophic < 0.2 mm	Two subtypes: - Intermediate 0.08–0.6 mm (10–40% mature) - Basaloid 0.1–0.5 mm (<10% mature)	- Papillary surface with deep and wide rete ridges - Koilocytes (multinuclear cells, irregular nuclei with halo, dyskeratosis)	- Thickened epithelium, smooth surface - Basaloid cells replace the full-thickness epithelium. - Worse prognosis than the warty subtype	- Combined features of warty and basaloid subtypes

**Table 6:** Histopathology characteristics of PeIN.

PeIN	Microscopic Findings	Immunohistochemical p16 Overexpression	HPV Association
**Differentiated**	Acanthosis, hyperkeratosis, hypergranulosis. Keratin pearls. Elongated rete ridges. Dyskeratosis. Absent atypia or koilocytes. Basal keratinocytes: eosinophilic, abundant cytoplasm; irregular, hyperchromatic nuclei; rare mitotic figures.	Absent.	Not HPV-driven. Lichen sclerosus-associated.
**Undifferentiated**	Parakeratosis. Dyskeratosis. Atypia, which affects most of the epidermis. Koilocytes. Pleomorphic cells. Three subtypes:	Positive	HPV-driven.
	**Warty:** spiking architecture. Intense cellular pleomorphism, hyperkeratosis, and parakeratosis.	Positive.	
	**Basaloid:** a flat architecture. Monomorphic small-sized, ovoid, basophilic cells with amphophilic cytoplasm, and evident nucleoli.	Most positive.	
	**Mixed (basaloid and warty)**	Positive.	
PEKMB	**Achantosis. Hyperkeratosis. Pseudoepitheliomatous hyperplasia.**	Negative.	Not HPV-driven. Lichen sclerosus-associated.

PEKMB: Pseudoepitheliomatous keratotic and micaceous balanitis.

## Data Availability

The raw data supporting the conclusions of this article will be made available by the authors upon request.
